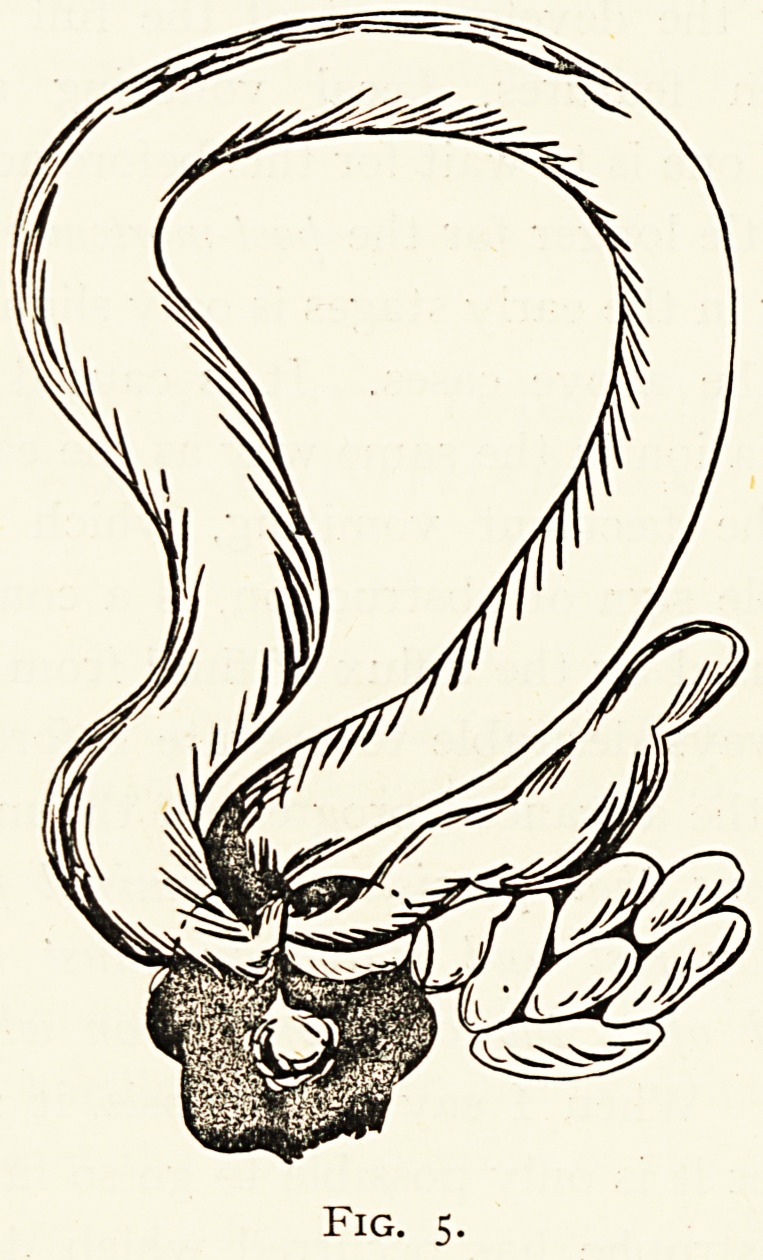# Some Cases Illustrating the Difficulty and Importance of Early Diagnosis in Acute Intestinal Obstruction

**Published:** 1912-03

**Authors:** Ernest W. Hey Groves

**Affiliations:** Assistant Surgeon to the Bristol General Hospital


### SOME CASES ILLUSTRATING THE DIFFICULTY AND IMPORTANCE OF EARLY DIAGNOSIS IN ACUTE INTESTINAL OBSTRUCTION.

BY

ERNEST W. HEY GROVES, M.S., F.R.C.S.,

*Assistant Surgeon to the Bristol General Hospital.*

---

IN forming a complete clinical picture of a typical case of intestinal obstruction, it is necessary to assemble all the signs and symptoms of this condition as forming parts of a whole; but it is of the utmost importance to remember that in the majority of cases diagnosis must be made and action must be taken, long before the picture is complete. We must be prepared to act upon a reasonable probability, and not wait for convincing demonstration in many surgical conditions, notably in acute abdominal disease and in the case of malignant growths. The following cases are all those of acute obstruction of the bowels which came under the writer's care within fifteen months, exclusive of those of strangulated hernias; and they illustrate in a striking manner two important points, viz. first, on what slender ground a diagnosis must often be founded, or rather how the exact diagnosis must often be deferred until after opening the abdomen, and second, what extreme changes occur in the intestine in a very short time.

**Case 1.**—Enteric intussusception (Fig. 1). S.H., aged 24, working man. Admitted to the Cossham Hospital June, 1910, for abdominal pain.

History.—No previous illness. Twenty hours before operation he felt a sudden pain in the abdomen just as he was rising from bed; this was followed by slight vomiting. The pain continued without much change, but the vomiting did not persist.

Condition.—Uniform distension of the abdomen, with some diffuse tenderness. Pulse 110, temperature normal. Bowels not open since the pain began.

Operation was decided upon at once because of the distension associated with persistent pain and constipation. Median incision, quantity of blood-stained exudate. A large coil of black gut, each limb being about one foot long and two inches in diameter, was easily brought to the surface. It consisted of an intussusception of the upper part of the jejunum, and the highest part of the superior mesenteric vessels was dragged into its neck. It was necessary to reduce the invagination in order to clear the neck from these important vessels. This was a matter of some difficulty, and in doing it the ensheathing layer was torn in several places. A piece of gut measuring

FIG. 1.

four feet nine inches in length was resected, this being about two inches more than the gangrenous portion. The cut ends of the gut were closed by purse-string sutures, and a lateral anastomosis formed.

The patient made a rapid and uneventful recovery. A peculiar fact was discovered in subsequent examination of the resected gut, viz. that a polypus about the size of a small walnut was growing from the mucous membrane in the neck of the entering layer. This was proved to be a simple adenoma. The existence of a polypus at the apex of an intussusception is, of course, well known to be a common cause of the condition ; but it is almost unique to find such a growth at the neck, and its relation to the invagination is difficult to surmise.

**Case 2.**—Strangulation under a band (Fig. 2). John S., aged 55, labourer. Admitted to the General Hospital June, 1910, for abdominal pain.

History.—No previous illness. Sudden pain in the abdomen twenty-eight hours before the operation. He was in the Hospital for twelve hours before the gravity of his condition was realised. During this time he had a little vomiting, but the bowels were opened after an enema. The temperature was

FIG. 2.

normal, but the pulse rose to 120, and the abdomen became slightly distended.

Operation was decided upon because of the steadily rising pulse rate and the abdominal pain and distension. Median incision. A quantity of foul-smelling, blood-stained fluid escaped, and a large distended coil of black gut was found strangulated under a long thin band, stretching from the right iliac fossa up to the right side of the mesentery. This band was as tight and as thin as a fiddle-string. The vessels in the imprisoned piece of intestine, which was a high part of the jejunum, were thrombosed, and the foul smell of the exudate proved that rapid transudation was taking place through its devitalised wall. The gangrenous gut, measuring a little over two feet in length, was resected by the cautery. A lateral anastomosis was done after closing the open ends of the bowel. The wound was drained. The patient made a rapid recovery, and has since been quite well.

**Case 3.**—Tuberculous peritonitis (Fig. 3). Mervyn T. aged 13, admitted to the General Hospital August, 1911, for severe abdominal pain.

History.—He had been healthy until one year previously, since which time he had had several severe attacks of

Fig. 3.

abdominal pain. On two occasions he had been admitted to the Hospital on this account, once under a physician and once under a surgeon, but both times he had been discharged because nothing definite could be detected which required active treatment. On the present occasion the pain has been unusually severe, but he has taken his food well and the bowels have acted regularly.

Condition (August 23rd, 1911).—A rather thin boy, in whom no objective evidence of disease could be discovered. The abdomen moves well, it is not tender, but rather full. The pain which occurs in paroxysms is very severe, and at night he keeps the other patients awake by his screams.

Operation was in this case determined on simply on account of the severe pain. Median para-umbilical incision. Some clear free fluid. Extensive tuberculous peritonitis, with many caseous glands in the mesentery. A tight band about half an inch wide, connected at one end to the right side of the mesentery and at the other to the lumbar parietes on the left side, constricted the jejunum about twelve inches from the duodenum. This was divided. The gut beneath the line of constriction was so thin and ecchymosed that it had to be sewn over. The boy made a good recovery.

In this case the absence of vomiting and constipation is especially noteworthy.

**Case 4.**—Strangulation by a Meckel's diverticulum. Lily W., aged 11. Admitted to the Cossham Hospital April, 1911, because of abdominal pain and vomiting.

History.—Had had chorea for some months. On April 26th she had sudden abdominal pain, but was not ill enough for the doctor to be sent for. Bowels acted with a normal motion. April 27th she was no better, the pain being more constant, and localised on the right side. Dr. Skelton saw her, and thought she had appendicitis. Admitted April 28th.

Condition.—Appeared to be very ill. Pulse 20, respiration 40, temperature 99°. Abdomen distended and tender, more so over the right iliac fossa, which region was also somewhat dull to percussion. Bowels acted slightly after a turpentine enema. Diagnosis of acute appendicitis.

Operation.—Appendix incision. Free blood-stained fluid. Some coils of distended black gut appeared, and it was necessary to make a median sub-umbilical incision. The lower end of the ileum was tightly strangulated by a cord, the thickness of a sash-cord, wound round the base of the affected loop. This being quite gangrenous was resected, forty inches in all being removed. The gut was re-united by an end-to-end union. The appendix was brought out through the wound, cut short, and used for continuous saline infusion. Her condition improved for about six hours after the operation. She then vomited a large quantity of blood-stained fluid, and died suddenly. There was no autopsy.

If the likelihood of acute intestinal obstruction had been suspected in this case, an early operation would have been performed, and the life might have been saved.

**Case 5.**—Strangulation by appendix and Meckel's diverticulum (Fig. 4). Raymond B., aged 6. Admitted to General Hospital September, 1911.

History.—No previous illness. On September 9th he woke up at 4 a.m. with sudden abdominal pain, which was followed by vomiting. This continued all day. The bowels were not open. On September 10th the vomiting had ceased, but the pain continued.

Condition.—Looked ill. Temperature 100°, pulse 120. Abdomen a little distended, some signs of free fluid, an indistinct mass felt in the right iliac fossa. Diagnosis was made of acute appendicitis, and immediate operation performed.

Operation (September 10th, 1911).—Appendix incision. Gangrenous gut presented. Mid-line incision. A short loop of ileum was tightly surrounded at its base by a cord consisting of an appendix which was sloughing, and a Meckel's diverticulum adherent to one another. The gangrenous gut (about six inches),

FIG. 4.

*The upper diagram in this figure shows the manner in which the appendix and the diverticulum first become adherent to one another so as to form a band which subsequently ensnares a loop of intestine.*

together with the appendix and the diverticulum, was resected, and an end-to-end junction made. A venous infusion of saline solution with pituitary extract was made at the end of the operation. Later in the evening a continuous subcutaneous saline infusion was carried out. He survived only about eighteen hours, dying in the afternoon of September 11th.

**Case 6** (Fig. 5).—Strangulation by the pedicle of a fibroid. Martha C., aged 71. Admitted to the General Hospital on September 10th, 1911, for abdominal pain.

History.—She had been told by Dr. Aust Lawrence twenty years ago that she had a "tumour of the womb," otherwise she has always had good health. Early in the morning of September 10th she was taken with severe abdominal pain, with a little sickness. The bowels had since been slightly opened.

Condition (eighteen hours after onset).—She had a drawn and anxious expression. Pulse 120. Temperature 97.4°. The abdomen was soft and not distended. There was slight general tenderness. An enlarged nodular uterus filled up the pelvis. Immediate operation.

Operation.—Sub-umbilical incision. A large quantity of almost pure blood escaped from the incision. A loop of ileum

FIG. 5.

was strangulated in the pelvis by the thin pedicle of a sub-peritoneal fibroid, turned back into Douglas's pouch. This fibroid pedicle was so slender that it broke on manipulation, but it had served to cause gangrene in the coil of incarcerated gut. This, amounting to about three feet, was resected, and a side-to-side anastomosis performed. The patient died in spite of a venous saline infusion performed on the table.

My special object in presenting this short series of consecutive cases is to illustrate and emphasise the difficulty and importance of making an early diagnosis in conditions of intestinal obstruction.

It will have been observed that the history in all of these was comparatively short (between eighteen hours and three days). Now it may be true that a case of intestinal obstruction will live for a whole week in the absence of any operative relief, but in the majority of cases the fate of the affected gut is determined, and immediate operation demanded within twenty-four to forty-eight hours.

It is utterly unjustifiable to wait in any case of suspected intestinal obstruction beyond this time. A longer period may be necessary for the development of the full clinical picture, with the sunken features, fæcal vomiting and abdominal distension, but if one is to wait for this before acting, one might as well wait a little longer for the *post-mortem* examination.

The vomiting in the early stages is only slight and transient, as is seen in all the above cases. It is caused by the sudden peritoneal stimulation in the same way as the early vomiting of appendicitis. The fæculent vomiting, which perhaps is the only unmistakable sign of obstruction, is a comparatively late phenomenon, caused by the reflux of fluid from the contracting bowel. It is always desirable to operate before the advent of this evidence of the advanced progress of the malady.

*It seems to me that persistent abdominal pain of a severe type, unrelieved by rest and starvation, must remain the most notable sign, and often the only one, upon which a diagnosis is to be founded.* When I say a diagnosis, it should be noted that in most cases it is only possible to go so far as to say that some grave catastrophe has occurred which demands further investigation. The case may turn out to be one of appendicitis, intestinal obstruction or pancreatitis, but it is better to "look and find out" than to "wait and see."

With certain precautions, the general rule that persistent abdominal pain, unrelieved by rest and starvation, requires the abdomen to be opened, will often be the means of saving life, though very rarely it may lead to an unnecessary operation. By persistent abdominal pain, in relation to the acute conditions we are considering, is meant pain with an abrupt onset in a patient who has been in good health, which persists for more than twenty-four hours, although the patient has been kept in bed on the lightest possible diet.

Such conditions as lead colic, tabes dorsalis, tuberculous spine and aneurysm must be borne in mind, and each can usually be excluded by the absence of characteristic signs.

The only condition likely to be mistaken for intestinal obstruction, in which operation will lead to disaster, is the acid intoxication of children. In this there is persistent vomiting, but there is a marked absence of pain, a tendency to drowsiness, and a sweet smell in the breath which should serve as warnings, which will be confirmed if the acetone bodies are found in the urine.

## Figures and Tables

**Fig. 1. f1:**
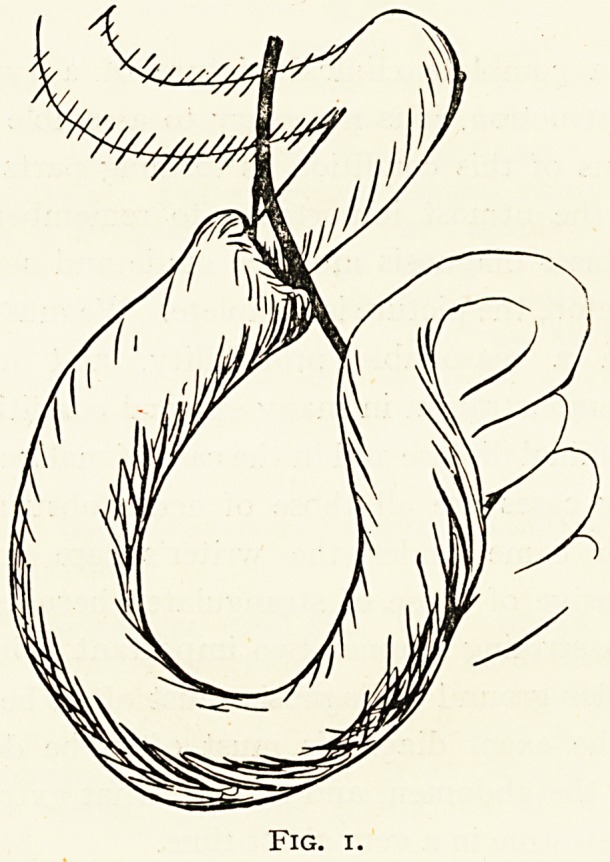


**Fig. 2. f2:**
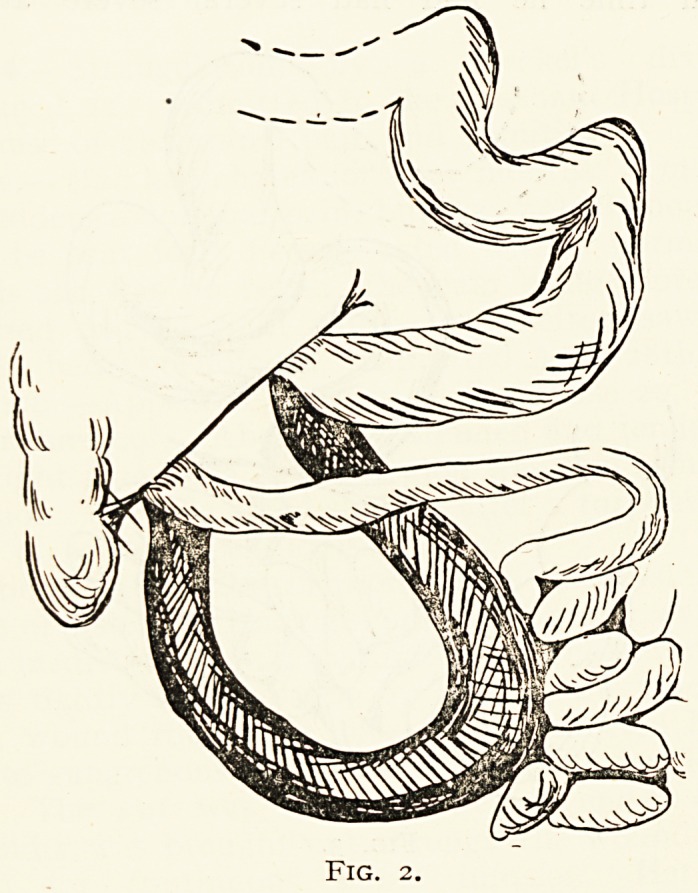


**Fig. 3. f3:**
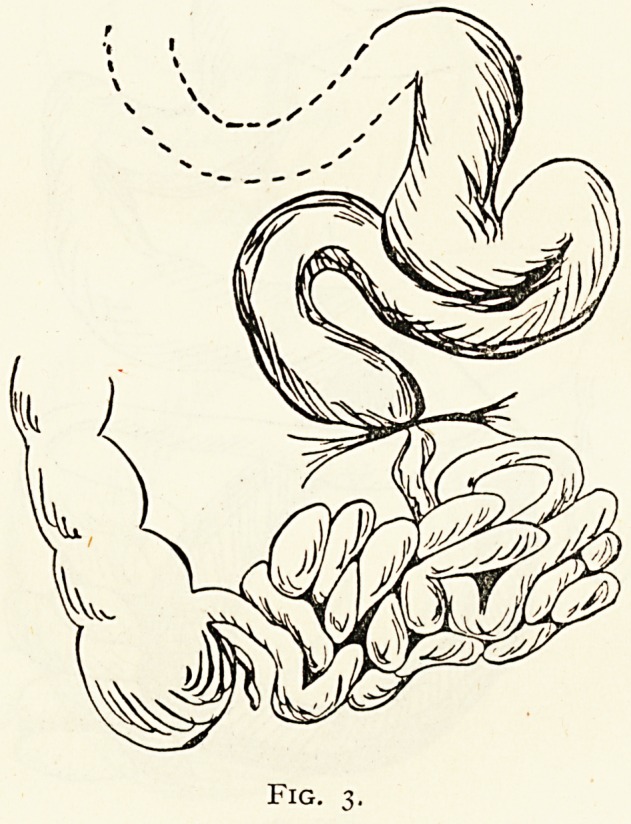


**Fig. 4. f4:**
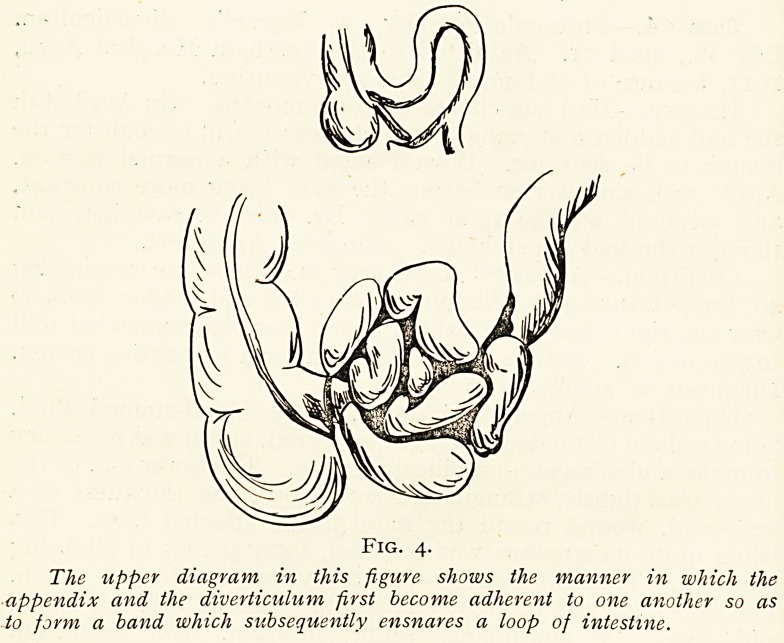


**Fig. 5. f5:**